# A rural community moves closer to sustainable obesity prevention - an exploration of community readiness pre and post a community-based participatory intervention

**DOI:** 10.1186/s12889-019-7644-x

**Published:** 2019-10-30

**Authors:** Jillian Whelan, Penelope Love, Lynne Millar, Steven Allender, Catherine Morley, Colin Bell

**Affiliations:** 1 Institute for Health Transformation, Global Obesity Centre, School of Health and Social Development, Deakin University, Geelong, Australia; 2 Institute for Physical Activity and Nutrition, School of Exercise and Nutrition Sciences, Deakin University, Geelong, Australia; 3 Adjunct Fellow, Victoria University, Melbourne, Australia; 4 Wimmera Health Care Group, Horsham, Victoria Australia

**Keywords:** Community readiness, Obesity prevention, Rural health inequity

## Abstract

**Background:**

Understanding levels of community readiness can result in prevention efforts that align with communities’ ability and capacity for change and, therefore, be more effective and sustainable. Our study aimed to use baseline (pre-intervention) community readiness scores to assist with the development of obesity prevention strategies, and to assess changes in community readiness over time (pre/post- intervention), to provide evidence of intervention impact.

**Method:**

Our study was located in a rural and remote area of Victoria, Australia. Community readiness was part of a broader obesity prevention intervention designed to create healthier food and physical activity environments through the combination of systems thinking and collaborative community-led solutions. Interviews were conducted using the community readiness to change tool in 2016 (pre) and 2018 (post) with a community representative sample. Baseline data informed the development of community relevant strategies and the pre/post results formed part of the overall evaluation.

**Results:**

The tool generated both quantitative and qualitative (quotes) data. A final readiness score was calculated that corresponded to one of the nine stages of readiness. Four of the five domains of the community readiness to change tool showed statistically significant improvements over time (*p* < 0.05): knowledge of effort, knowledge of issue, community climate, and leadership. The resources domain that did not improve pre/post intervention.

**Conclusion:**

Community readiness to change interviews, pre- and post- intervention, provided essential information related to the appropriate targeting and pitch of the prevention strategies, as well as providing an overall evaluation of the positive movement in the community’s readiness to implement change.

## Background

Public health practitioners work with communities to mobilise prevention efforts. These efforts are most effective when tailored to the local context and when communities are empowered to make change [1]. To address the complex nature of public health problems, co-design (practitioner and community) and systems thinking are replacing discrete projects that previously adopted linear logic models [2, 3]. Such co-designed systems approaches depend heavily on the capacity and readiness of the community to lead and action change [4]. It is therefore imperative to measure this readiness so the intervention can be tailored appropriately, helping to achieve maximum engagement and impact for each community at their stage of readiness.

Several methods of assessing readiness to change have been published. At an individual level, the trans-theoretical model of behaviour change is commonly used to explain a five stage process of change [5, 6]: pre-contemplation, contemplation, preparation, action and maintenance [7]. Specific tools to assess readiness to address particular health problems, such as eating disorders, have also been developed [8]. Beyond individual change strategies, setting specific tools have also been developed, such as school readiness [9], or organisational level tools [10], to measure readiness through emotional, cognitive and intentions dimensions.

Organisational and personal behaviour change models/tools do not adequately match the change cycle of a community. For example, community level interactions, community resources, leadership, and environments all play a part in community change and are beyond the control of any one individual, one organisation or one setting. Community readiness is a merger of individual psychological readiness and community development principles, defined as the ‘degree to which a community is willing and prepared to take action on an issue’ [11] p.4, and ‘the observable and psychological characteristics of a community that influence its ability to initiate change’ [4] p. 2. The Tri-ethnic Center for Prevention Research developed the first standard methodology (Community Readiness Tool (CRT)) for describing and assessing community readiness [11]. The CRT has been used to help communities address a range of public health problems including tobacco control [12], HIV [13] and obesity [14–16].

The CRT has also been applied in a variety of ways: to guide decision making on funding interventions [4]; measure readiness to change in disadvantaged communities [17]; pre-post intervention changes in readiness [15, 18], and to assist in the strategic targeting of intervention messaging. It has also proven to be a useful tool in the evaluation of complex interventions as it assesses the problem from multiple perspectives across multiple domains [19]. The CRT has been used in studies conducted in high, middle and low income nations including USA [20], Canada, United Kingdom [21], India, Bangladesh and Australia [15, 17].

In our study we used the CRT to inform and evaluate the impact of a whole of community, systems level obesity prevention initiative (known as YCHANGe) implemented in a rural community in Victoria, Australia [22]. We hypothesised that a better understanding of the level of community readiness would enable us to tailor intervention strategies to meet differing needs across the community. We also hypothesised that our intervention would improve the level of community readiness to change over time.

## Methods

We followed the reporting recommendations for primary studies which apply the CRT [19].

### Context of application

This study was conducted in the rural and remote community of Yarriambiack Shire Council in Victoria, with a population of 6673 [23] spread across 7158 km^2^. At the time the study commenced, Yarriambiack experienced adult prevalence of overweight and obesity 13.6% above the Victorian average and had the highest per capita intake of sugar-sweetened beverages of all Victorian local governments [24]. The current study was instigated by the local health service, Rural Northwest Health [25], with the long-term goal to reduce avoidable hospital admissions. A coalition of local stakeholders evolved into a collaboration of key leaders and community members, including the research university, with the aim of creating healthier food and physical activity environments. For the purposes of our study, ‘Community’ was defined in two ways: (i) the overarching definition of ‘community’ was the geographical boundary of the local government. Although one administrative area, community members identified as three distinct communities, predominantly due to historical geographic boundaries, which are described in this paper as the north, central and south [11]; and (ii) we defined ‘community’ relevant to the context or setting to be captured, (explained further in the participants section). While data were captured separately across the three geographical boundaries and by settings, due to anonymity requirements results are reported for the local government as a whole.

### Objectives

The primary objective of this study was to:

1. Use baseline (pre- intervention) community readiness scores to assist with appropriate targeting of obesity prevention strategies.

The secondary objective of this study was to:

2. Assess changes in community readiness over time, at baseline and follow-up (pre- and post- intervention) to inform overall impact of the intervention.

### Participants

CRT suggests interviewing between 6 and 10 participants for each community [11]. At baseline in 2016, we utilised purposive sampling to obtain representation from the following sectors or settings in each of these three geographic communities (north, central, south): health; local government; early years; schools; workplaces; service clubs; sports clubs; general community members (holding multiple community roles), an average of 8 participants per community. Eligible participants were over 18 years of age and either lived or worked in the community. Some participants had employment that reached across the whole local government and therefore were able to report on the three geographic communities, others answered specifically to their local geography and/or their relevant context or setting. Baseline participants were followed up in 2018 if they still resided or worked in the community. Ethics approval was obtained from Deakin University (HEAG-H 80_2016).

### Data collection

The validated CRT tool [26] was used to collect information via structured interviews with key community stakeholders pre- and post- the obesity prevention intervention, following guidelines for adaptation of the CRT to the relevant issue under investigation [11]. All interviews were conducted in person by JW or PL. The CRT was downloaded onto electronic tablets and responses were entered directly into the tablet at the time of the interview. Interviews lasted between 20 and 60 min. Informed consent was collected at the commencement of all interviews.

The interview schedule comprised five sections corresponding to the five CRT domains [11]: community knowledge of the issue; knowledge of the efforts; community climate; leadership; and resources; with 42 ‘anchored’ questions and 19 open-ended text responses questions. The issue to be addressed was described as ‘overweight and obesity within your community’. We adapted the wording, as per CRT protocol, to address overweight and obesity and asked participants to answer the questions from the perspective of the community they know best, not from their personal viewpoint.

### Data analysis

The interviews were scored using descriptive statements on anchored scales [11] and awarded a final community readiness score per domain and overall [27]. This final score corresponded to one of nine stages of community readiness [11] as shown in Table 1.

**Table 1:** Nine stages of community readiness from the Community Readiness Tool (adapted from Oetting et al. [11])

Stage #	Stage Title	Description
1	No awareness	Issue is not generally recognized by the community or leaders as a problem (or it may truly not be an issue).
2	Denial/Resistance	At least some community members recognize that it is a concern, but there is little recognition that it might be occurring locally and not support to provide resources to address the issue
3	Vague awareness	Some feel that there is a local concern, but there is no immediate motivation to do anything about it.
4	Preplanning	There is clear recognition that something should be done, and there may even be a group addressing it. However, efforts are not focused or detailed.
5	Preparation	Active leaders begin planning in earnest. Community offers modest support of efforts.
6	Initiation	Enough information is available to justify efforts. Activities are underway and some resources exist.
7	Stabilisation	Activities are supported by community leadership, administrators or community decision makers. Staff are trained and experienced.
8	Expansion/Confirmation	Leadership plays a key role, majority of community strongly support action, considerable allocated resources.
9	Community ownership	Most community members have considerable and detailed knowledge of efforts, leadership is highly engaged, diversified resources and funds are secured.

CRT scoring was conducted by two researchers independently (JW and CB) using the scoring tool and protocol provided [11]. Individual scores were then compared and discussed until consensus was reached. The mean and standard deviation (SD) for each domain was calculated separately, reported per domain. The overall community readiness score was calculated by averaging the five domain scores. Descriptive statistics and Wilcoxon signed-rank tests were conducted in SPSS version 25 [28]. Wilcoxon signed-rank tests were conducted to test significance of differences between baseline and follow-up scores for each domain and overall. Results were considered statistically significant at *p* < 0.05. Open-ended responses were entered into NVivo and themed according to the five domains of the CRT. Relevant quotes are presented where they add context to the results.

## Results

### Participants

We surveyed 28 purposefully selected community stakeholders at baseline and 22 at follow-up. No demographic data were collected. Six fewer interviews were conducted at follow-up due to staff turnover (*n* = 2), staff leave (*n* = 1), restructuring of agencies thereby reducing overall positions (two health services amalgamated into one (*n* = 1), illness (*n* = 1) and one non-response (*n* = 1). After reviewing the profile of follow-up participants, researchers agreed that adequate and comparable community responses had been captured. On analysis of the follow-up open-ended responses, it was considered that no new themes were emerging, therefore data saturation had been reached and it was deemed unlikely that new viewpoints could be obtained beyond the recorded non-responses.

### Community readiness data

Results are presented to address the two primary objectives of this study. Firstly we used the baseline scores to assist with strategy design. Table 2 shows the baseline CRT scores across each domain and examples of strategies implemented in the intervention to shift the community to a higher level of readiness, for example extensive media to raise knowledge of the issue and of the efforts, multi-stakeholder backbone to engage broad leadership.

**Table 2:** Strategies used to target baseline low levels of readiness in YCHANGe

Community Readiness Model Dimensions (Definition)	Baseline Readiness Score	YCHANGe strategy	Strategies to increase readiness levels
Knowledge of issue	3.61 (vague awareness)	Media Social marketing and social media	Weekly newspaper articles School newsletter articles Presentations at AGMs and community groups Radio interviews Attendance at local community events Facebook (126 followers) Twitter YCHANGe branded resources (water bottles, t-shirts, placecards) Establishment of YarriYak Café
Knowledge of efforts	3.13 (vague awareness)		
Community Climate	3.39 (vague awareness)		
Leadership	4.25 (preplanning)	Meetings with key stakeholders	Individual and small group meetings with CEO and executive management leaders across the area.
		Establish backbone organisation	Multi-stakeholder backbone with CEO representation from seven local organisations and the mayor as community representative met three times per year.
		Establish steering committee	Steering Committee: comprised of representation from the following sectors: health, education, disability, neighbourhood houses, sports and general community representatives met six times per year.
		Community champions	Working groups met monthly
Resources	3.07 (vague awareness)	Mobilise existing employed resources in health promotion and community development.	Face to face meetings with relevant CEOs and executive level management.
		Engage with GPs to prioritise prevention.	Training for General Practice Nurses on prevention models and maximising business case for prevention.
		Meetings with key stakeholders in local government to re-orient existing policy.	Policy changes to incorporate health and wellbeing in LGA strategic plan.
		Training for local community members in healthy eating	Partnerships with health promotion expertise.
		Capacity building through training of employed staff and volunteers	Community training on healthy choice food guidelines.
			Community training on understanding systems thinking and complexity.
		Short term grant funding to mobilise efforts	Various government, philanthropic sources and funding from Rural Northwest Health

Secondly, we measured follow up CRT scores and compared these to baseline scores for each CRT domain and overall. Table 3 lists each domain with the relevant question and results.

**Table 3:** Mean and standard deviation scores for baseline and follow-up readiness to change domain

Domains	Baseline		Follow-up		Change	*P*
	M	SD	M	SD		
Knowledge of issue ‘How much does the community know about the issue?’	3.61	1.46	5.57	1.87	+ 1.96	< 0.001
Knowledge of efforts ‘How much does the community know about the current programs and activities?’	3.13	1.49	6.25	2.25	+ 3.12	< 0.001
Community climate ‘What is the community’s attitude toward addressing the issue?’	3.39	0.92	5.02	1.69	+ 1.63	0.002
Leadership ‘What is leadership’s attitude toward addressing the issue?	4.25	1.01	5.20	2.01	+ 0.95	0.032
Resources ‘What are the resources that are being used or could be used to address the issue?	3.07	0.86	3.16	1.00	+ 0.09	0.868
Overall	3.49		5.04		+ 1.55	< 0.001

Most baseline scores were in the range of ‘3’, which represents ‘vague awareness’, with leadership in the ‘preplanning’ phase. No community stakeholders identified ‘no awareness’ or ‘denial/resistance’ to the issue. Follow-up scores (except resources) were significantly higher than baseline; the increases ranged from 0.95 to 3.12 units on the scale.

*Knowledge of Issue* increased by 1.96 units (*p* < 0.001), shifting the readiness score from a state of ‘vague awareness’ to ‘preparation’. This significant improvement was frequently aligned with YCHANGe communication strategies, for example: pre-intervention, a community member was: ‘*… unaware of any information that is readily available to the public*’ whereas post-intervention, comments echoed a much stronger awareness *‘through the media, children coming home from school and kinders’.* Despite this positive shift in knowledge, there remained a concern about a lack of understanding of the extent and/or the true complexity of obesity: *‘[community] don’t understand the complexity of the issue and the solutions, regular access to quality fresh produce is a problem’.*

*Knowledge of Efforts* increased by 3.12 units (*p* < 0.001), shifting the readiness score from a state of ‘vague awareness’ to ‘initiation’. Comments from participants pre-intervention included: ‘*poor marketing of the events that are on and a lack of support from the general population*’; ‘*lack of interest’*, and ‘*not a lot of the community investigate or ask questions about the activities*’. Follow-up responses were consistent with an increase in knowledge: *‘those who have engaged know the benefits, then shared their stories with other community members’.* YCHANGe appeared to be visible with comments such as: *‘water fountains are really popular’*, ‘*healthy food in the cafeteria now’* and *‘limited sweet cold drinks in the machines’*.

*Community Climate* increased by 1.63 units (*p* = 0.002), shifting the readiness score from a state of ‘vague awareness’ to ‘preparation’. Challenges faced by the rural farming community at the time were evident in their responses. Pre-intervention sentiment was summed up as ‘*There’s no key driving force within the community about obesity*’; at follow-up difficulties remained: *‘… focussing on: for example, income, families, costs/cropping, mental health, looking out for mates… the drought, overheads, keeping afloat with family, debt, bank, machinery.’* and *‘(there is a) lack of understanding of nutrition, food groups, cooking healthily’.* Post intervention, more stakeholders made positive comments such as *‘healthy eating is no longer hammered in words only, it is in practice’.* Further work is required to continue to improve community climate:*‘some community members are not interested perhaps they don’t see the long term benefits’.*

*Leadership* increased by 0.95 units (*p* = 0.032), shifting the readiness score from a state of ‘preplanning’ to ‘preparation’. Respondents highlighted the importance of ‘*influencers - people who … have respect in the community, people listen to what they say, they have more effect than people in positions’.* Responses to how leadership viewed obesity prevention varied substantially. Some respondents regarded leadership to be lacking or obstructive: *‘Leadership is very poor in these communities, [they] fail to work hard on any issue, ‘can’t rock the boat’,* and *‘Some school teachers/principals, some pub owners …show their opposition by declining/ignoring/not implementing suggestions made for healthy changes’.* Some respondents also identified leadership changeover as a problem *‘[The] new CEO has been here three or four months’*. Some others key leaders who *‘display great passion in the work they do to promote healthy changes’.*

The *Resources* domain did not show significant increase, with no shift in readiness from a state of ‘vague awareness’. Some respondents regarded the limited resources available within the community as a constraint to action: *‘not funded, no continuation’, ‘It comes back to resources. The end of YCHANGe is sad’*. Others respondents were concerned about the lack of continuity due to limited resources: ‘*We’ve started but don’t know how to follow through. We’ve laid the ground rules, who now takes the baton?’*

**Fig. 1:**
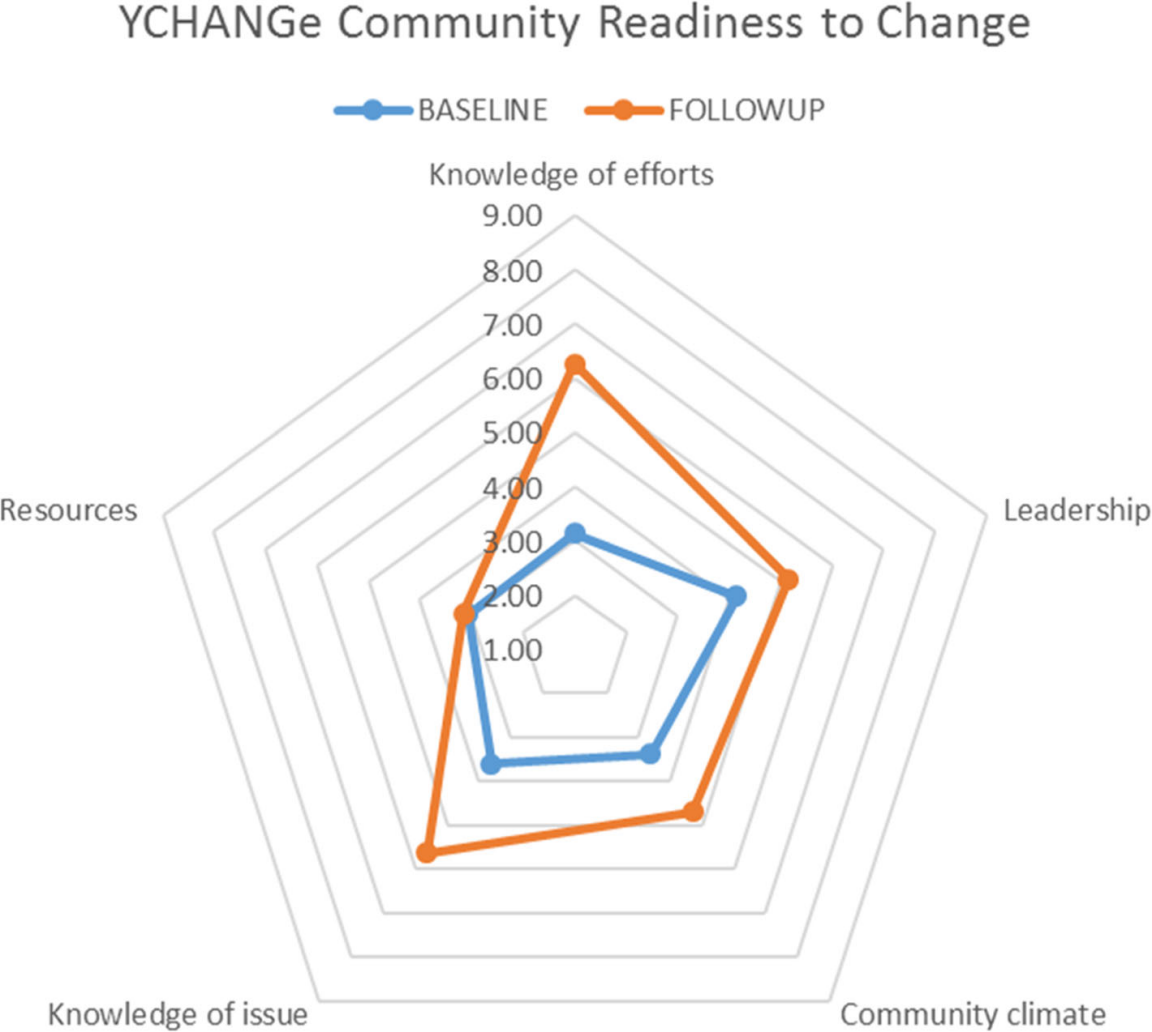
Baseline and followup community readiness domains

The aggregated readiness to change score showed a significant increase pre- and post-intervention by 1.55 units (*p* < 0.001), showing a shift in readiness from a state of ‘vague awareness’ to ‘preparation’. As described by one participant: *‘Our whole community is more considerate of healthier eating and active lifestyle - it is continually referred back to at staff meetings and parent meetings’.* Figure 1 shows baseline and follow-up community readiness to change scores.

## Discussion

### Interpretation

We set out to assess whether the community readiness to address obesity prevention in Yarriambiack Shire Council had improved between June 2016 and June 2018. Our first objective was to use the readiness scores to assist in the appropriate tailoring of community-relevant, obesity prevention strategies. Our second objective was to improve, over the time of the intervention, the readiness of the community to prevent obesity.

Our first objective led to the implementation of YCHANGe strategies as noted in Table 2, with the emphasis on moving the community to higher levels of readiness. The results reflect this was achieved in four of the five measured domains. Some of the strategies targeted awareness raising, whereas others were adopted at policy and leadership level and implemented. These responses varied according to the level of readiness of the organisation and particularly the leadership within organisations and within community groups. There were some signs of settings-based, systems change with the local health service a prime example. The health service put in place policy changes (healthy catering policy, traffic light system in the in-house cafe), environmental changes (removal of soft drinks from catering and from vending machines, an exercise path specific to the needs of the elderly and people with disabilities), as well as working with staff on individual behavioural change.

The second objective was to increase community readiness over the course of the intervention. As reported, increases were achieved in four of the five CRT domains. At baseline, the community was at stage 3 of a 9-stage readiness to change process, described as: ‘Vague Awareness’: ‘Something should probably be done, but what? Maybe someone else will work on this.’ [11] p. 7. At follow-up, the community was at the lower end of stage 5, the Preparation Stage which the CRT equates to ‘I will meet with our funder tomorrow.’ [11] p. 8. This aligns with our observation that active leaders were working earnestly to promote healthier food and physical activity environments, other leaders were not promoting change, despite their perceived power to do so.

This shift in readiness is promising but, given that the resources domain remained low and there were no long-term funders identified during the two-year intervention phase, further examination of readiness is required to see if the upward impetus will continue or the positive increases maintained.

### Generalisability

The purpose of undertaking CRT for this intervention was to utilise a community collaborative process to identify and implement readiness relevant strategies. We found that after 2 years of activity, there was a statistically significant increase in most dimensions of readiness even though follow-up scores were still relatively low and just reaching the preparation stage. Although moderate, the improvements achieved were realised with no additional resources, beyond the researchers within the community. The emphasis on creating a collaborative approach and a common agenda ‘to make the healthy choice the easy choice’ resonated well with the community, as assessed by the follow-up measures. For these reasons we consider the use of CRT to inform relevant community level interventions could result in improvements in readiness over time in other similar rural communities given the same opportunities.

### Overall evidence

Our overall score of 3.47 units (SD 0.80) (vague awareness) at baseline indicated that strategies aimed at raising awareness within the community were required. Our results are comparable to other baseline studies, such as the 3.06 readiness score for childhood obesity in rural USA [29], or a score of 4.0 by Cyril et al. [17] across four disadvantaged urban communities in Australia. This suggests that the tool can provide relatively consistent findings across urban and rural disadvantaged communities. As in other studies [20, 29], the strategies prioritised by community members were reviewed against baseline readiness to change and adjusted to fit the readiness of the community and community stakeholders.

Sliwa et al. [4] reported that readiness scores for leadership ≤3.9 adversely impacted funding applications for prevention interventions. Our average leadership baseline score of 4.1, although low, showed wide variation around the mean (SD 1.01), indicates that community perceives there to be different levels of leadership evident across the local government and in different sectors. A key example of this was differing approaches to a community-prioritised strategy to reduce the availability of sugary drinks within workplaces. In one workplace, the CEO proceeded, through awareness raising and policy implementation, to remove the availability of sugary drinks in accordance with the community priority, the current evidence and government guidelines [30]. In a second workplace, the CEO chose to survey their staff prior to action which resulted in a sign being placed on the refrigerator to provide information about sugar content of the drinks. Effective leadership has been linked elsewhere to sustainable obesity prevention [31] and the capacity to implement health promoting strategies through community diffusion [32].

In line with other studies that have measured readiness pre- and post- intervention, our study showed readiness to change improved over time across most domains [18]. Millar et al. [15] in an obesity prevention intervention in a regional area of disadvantage correlated increased readiness scores with a decrease in obesity prevalence. Although we do not have the appropriate data to assess BMI changes in this study, we have identified elsewhere positive behaviour changes [33].

Our overall CRT score at follow-up had increased to 5.04 units (SD 1.30), this was still under the threshold for initiation (a score of ≤6), and indicates more time is required to ready this community for sustainable change [4] and longer time frames generally for prevention to become sustainable [31]. We consider that the broader community-wide obesity prevention initiative (YCHANGe) played a role in increasing community readiness to act on obesity prevention as it was highly visible in the media and community events [34, 35]. There were no other obesity prevention initiatives in operation within the community at the same time. We synergised efforts with related community-action research projects and worked across organisations to reinforce messages of healthy living and healthy habits.

It is problematic that the resources domain (defined as human, financial and community facilities), did not improve from a state of ‘limited resources’, particularly in light of the systematic review on obesity prevention that highlights resources (human and financial) as the most frequently cited determinant of sustainable obesity prevention [31]. A low score on resources has also been reported in other areas of disadvantage in Australia and overseas [17, 18]. Due to limited human resources, this intervention relied heavily on community volunteers, especially retired community members. As observed by Munoz et al. (2014) [36], older rural volunteers are already over-stretched and may not have the capacity to take on any more activities. An understanding of a minimal level of resources required for sustainable change would further assist communities to assess their readiness to change.

We consider the CRT worked well to assess changes in community readiness in this relatively small community (*n* = <7000) as it was possible for the researchers to identify and reach key stakeholders. As noted elsewhere [17], use of the tool in larger communities would be time and resource intensive.

### Limitations and strengths

The researchers who conducted the CRT interviews were involved to varying degrees in the design and implementation of the overarching YCHANGe study, so it is possible participants exaggerated responses because of social desirability bias. One of the scorers (CB) played no role in the implementation of the study and therefore the scoring process was not subject to the same potential bias. We did not have information on community readiness from comparison communities meaning, although promising, we cannot claim YCHANGe is the sole reason for the change.

Although we scheduled interviews to suit the participants wherever possible, the time involved in undertaking the interviews may have been an issue for the non-responder. Turnover in rural communities proved a barrier to collection at follow-up. However, protocols were followed to ensure this minimised disruption to the final scores [11].

A strength of the study is the two-year follow-up measure to assess movements in readiness across the community. A further strength is the alignment of the implemented strategies to the level of readiness, which may have contributed to the community as a whole moving to a higher level of readiness to change.

### Implications for practice

We acknowledge the efforts of Kostadinov et al. [37, 38] to create an online version of the CRT, currently validated only for the leadership domain. Further research to validate this online forum across all other domains could broaden the reach of the tool and reduce time in interviewing and scoring.

In order to develop a sustainable obesity prevention intervention, it is essential to work with the community at their level of readiness. By assessing readiness, consulting with the community about priority areas and adjusting strategies to enhance readiness to change has potential to improve the long-term viability of prevention efforts. We contend that some level of minimum baseline resources is necessary for sustainable prevention.

### Future research

Further research is required to develop more time efficient means of collecting and analysing readiness to change data, particularly in rural communities where internet access remains unreliable.

Further follow-up on readiness to change should be conducted within this rural community to see if the improvements are sustained and built upon in a further 12 months.

## Conclusion

The conduct of pre- and post- readiness to change interviews provided valuable information a) to enhance baseline levels of readiness to change through the development of strategies targeted to the assessed level of readiness and b) to measure change in readiness over time; and a valuable adjunct to the overall evaluation of a whole of community obesity prevention initiative. It also reinforced findings that where resources are limited (both human and financial), the progress of obesity prevention activities is hindered.

## Data Availability

De-identified data is available at https://www.synapse.org/#!Synapse:syn18880256/files/. To access the data, a synapse account can be set up at no cost via: https://www.synapse.org/. Alternatively, the data is available on request from the corresponding author.

## Abbreviations

CRT: Community Readiness Tool
SD: Standard deviation
SPSS: Statistical Package for Social Sciences
YCHANGe: Yarriambiack – Creating Healthy, Active, Nourished Generations
